# Performance indicators on long-term care for older people in 43 high- and middle-income countries: literature review, web search and expert consultation

**DOI:** 10.1186/s12913-025-12573-4

**Published:** 2025-03-28

**Authors:** Mircha Poldrugovac, Joost D. Wammes, Véronique L. L. C. Bos, Erica Barbazza, Damir Ivanković, Hanneke Merten, Janet L. MacNeil Vroomen, Niek S. Klazinga, Dionne S. Kringos

**Affiliations:** 1 Department of Public and Occupational Health, Amsterdam UMC Location University of Amsterdam, Amsterdam, the Netherlands; 2 National Institute of Public Health of Slovenia, Ljubljana, Slovenia; 3 Department of Internal Medicine, Section Geriatrics, Amsterdam UMC Location University of Amsterdam, Amsterdam, the Netherlands; 4 Aging & Later Life, Amsterdam Public Health research institute, Amsterdam, the Netherlands; 5 Quality of Care, Amsterdam Public Health research institute, Amsterdam, The Netherlands

**Keywords:** Long-term care, Performance indicators, International comparisons, Pressure ulcers, Falls, Use of restraints, Pain management.

## Abstract

**Background:**

Long-term care (LTC) for older people is an area of focus for many health and social policies in high- and middle-income countries. Performance Indicators are used to provide national and subnational jurisdictions with insights to ensure quality of the provided LTC services for older people. Although LTC systems vary across jurisdictions, there is demand for internationally comparable indicators to support countries in monitoring LTC and facilitate mutual learning. The aim of this study was to provide an overview of indicators currently employed to monitor the performance of LTC systems and services in high- and middle- income countries and describe their key characteristics.

**Methods:**

A review of the literature in six scientific databases (literature review) and web searches of relevant sites across 43 selected countries (web search) was conducted. We asked country representatives from the Working Party on Health Care Quality and Outcomes of the Organization for Economic Cooperation and Development, where most of these countries are represented, to cross-validate the sources of information found (expert consultation). We then extracted and analysed the data from all obtained sources based on a predetermined set of characteristics.

**Results:**

The search of scientific databases yielded 12,960 records, from which forty papers were selected for inclusion. The scientific literature findings were complemented by 34 grey literature sources. In total, we identified performance indicators being used to monitor LTC systems and services across 29 national and subnational jurisdictions in 24 out of 43 countries. In total, 620 indicators were identified. All jurisdictions used indicators related to institutional LTC and 16 also used indicators on home care. The most frequently monitored structures, processes, and results were pressure ulcers, falls, use of restraints and pain management.

**Conclusions:**

We identified LTC performance indicators currently being monitored in 29 jurisdictions across 24 countries. Many jurisdictions are monitoring similar structures, processes, and results. This presents an opportunity to develop internationally comparable LTC performance indicators based on existing efforts across countries.

**Supplementary Information:**

The online version contains supplementary material available at 10.1186/s12913-025-12573-4.

## Background

Population ageing in high- and middle-income countries will have a profound impact on societies in the coming decades, necessitating substantial adaptations in health and care systems. Long-term care (LTC), in particular, faces escalating demand due to ageing populations and the rising prevalence of chronic conditions such as diabetes, chronic obstructive pulmonary disease, chronic heart failure, and dementia, as well as limitations with respect to mobility, hearing and eyesight. Projections indicate substantial increases in LTC expenditures and a growing demand for health care workforce resources [1]. For example, public expenditure on LTC in the European Union is projected to increase from 1,7% of GDP in 2022 to 1,9% in 2030 and 2,6% in 2070 [2].

Individuals with chronic illnesses, physical disabilities or frailties rely heavily on LTC services to maintain or achieve a good quality of life. However, LTC remains a sector with limited resources in many high- and middle-income countries [3, 4], despite its responsibility for a growing number of individuals in our societies facing complex care needs.

The United Nations and the World Health Organization have recognized the importance of health and care for older people by adopting the UN Decade of Health Ageing Plan of Action (2021–2030) [5]. The plan calls for the provision of long-term care to older people, including “monitoring of quality of long-term care” [5]. Assessing the nature and growth of the LTC sector is crucial for evaluating health system performance [2, 6, 7]. Currently, international performance information remains limited to exploratory studies focussing on areas such as the prevalence of pressure sores, medication use, healthcare associated infections, patient-reported experience measures (PREMS), patient falls and hospital referrals [6] and multi-country surveys such as the Survey of Health, Ageing and Retirement in Europe [8]. Given the increasing importance of LTC, it is essential to develop, report and apply appropriate performance indicators to inform improvements in care quality. Ideally, these performance indicators are internationally comparable [9–11]. By performance indicators we mean quantitative measures of performance, where performance includes the domains of safety, effectiveness, person centredness, efficiency, equity, timeliness, and integration of care. In an international context, this definition may be synonymous with quality [12] or broader than quality, incorporating additional dimensions beyond quality (as used by OECD) [13].

The organization of LTC varies widely across countries, encompassing diverse health and social policies, funding sources, and data-systems making direct cross-country comparisons challenging [7, 14]. LTC services are delivered in various settings, including a recipient’s home, community centres, assisted living facilities, nursing homes and hospitals The Organization for Economic Cooperation and Development (OECD) has developed the System of Health Accounts, which is used by its member countries also to report on spending on LTC [15], thus providing a common understanding of LTC. Based on the OECD system, spending on LTC are categorized as either health or social care related. Among the healthcare related spending, most are due to either inpatient LTC or home-based LTC.

There is a substantial body of knowledge regarding potential performance indicators for monitoring LTC, as well as indicators developed and used in specific studies. However, existing reviews often focus on specific LTC settings or conditions [16–18]. National and subnational jurisdictions have official authorities with responsibilities for monitoring a jurisdiction’s LTC systems and services performance, such as ministries of health and social care or public agencies responsible for quality or LTC. As these authorities aim to establish LTC performance monitoring, they encounter a number of implementation challenges. While the scientific soundness of an indicator is crucial, it alone does not guarantee its effective integration in regular monitoring practices [19]. The availability of reliable data sources and the necessary infrastructure for data collection present major implementation hurdles [20, 21]. It is, therefore, important to investigate which performance indicators national and subnational authorities have actually been able to implement and are currently using to monitor the performance of their LTC systems. Few studies address this specific focus [22].

Improving our understanding of the state of the art in monitoring LTC performance across national and subnational jurisdictions by official authorities is needed to identify commonly monitored aspects, assess feasibility, and establish a basis for developing internationally comparable performance indicators. This study aims to contribute to fulfilling these needs by addressing the following questions:

1. What indicators are employed to monitor the performance of LTC systems and services in high- and middle-income countries?
2. What are the key characteristics of these indicators in terms of the domains covered, the structures, processes, and results monitored and the LTC settings under scrutiny?

## Methods

We designed this study using a scoping review approach, following the framework proposed by Arksey and O’Malley [23] and enhanced by Peters et al. [24–26]. The review protocol was registered with the Open Science Framework in July 2019 [27], after completing the first round of database searches and before commencing paper selection and hand searches.

Initially, our search strategy relied on scientific database searches and hand searches of non-indexed grey literature including web-based materials. However, as the study progressed, it became evident that the most up-to-date information on performance monitoring on LTC could predominantly be found through grey literature, in particular in web-based materials. Consequently, after conducting a thorough scientific literature review, our approach deviated from the registered protocol as data extraction focused on web-based material. We report on the Preferred Reporting Items for Systematic reviews and Meta-Analyses extension for Scoping Reviews (PRISMA-ScR) Checklist [28] in Additional file 1.

### Database search

We systematically searched six scientific databases: Embase, Medline, PsycINFO, Cochrane, CINAHL and Web of Science. The initial search was conducted in April 2019. To ensure up-to-date results, we repeated the search in April 2022, using the same methodology, but restricting the publication period to studies published between April 2019 and April 2022. The search strategy was designed around the concepts of “performance framework” and “LTC”, using both MeSH terms and natural language. The natural language search included terms related to “performance”, its domains, “PREMS” and “PROMS” and the proximity of these terms to concepts similar to “framework”. Additionally, we searched for long-term care, specific long-term care settings and combinations of concepts related to “care” and “old persons”. The term framework was used to make sure that publications addressed efforts to monitor LTC systems and services comprehensively, as opposed to single indicators used in a narrowly defined context. An example of the search strategy used in Medline (OVID) is provided in Additional file 2.

### Screening of papers

The inclusion and exclusion criteria were established based on the registered protocol. To ensure consistency in applying these criteria, we initially screened 217 titles selected from the database search results. The screening process and results were then discussed among all authors to calibrate the interpretation and application of the criteria. Following this calibration process, two additional inclusion criteria were introduced: a requirement for a specific focus on performance frameworks related to older persons and a requirement that retrieved papers be original research (Additional file 3). Subsequently, a two-pass verification method for the screening process for titles, abstracts and full papers was conducted in researcher pairs, with disagreements resolved through discussion and, when necessary, involvement of other authors. The screening was performed using Clarivate EndNote 21.2 and Microsoft Excel version 2402 and the Rayyan online tool (rayyan.ai).

### Hand search

The hand search involved reviewing online resources across OECD member countries [29] and countries in the WHO European Region [30] that reported having LTC data to WHO in a recent survey A total of 43 countries were reviewed, including Australia, Austria, Belarus, Belgium, Canada, Chile, Colombia, Costa Rica, Czechia, Denmark, Estonia, Finland, France, Germany, Greece, Hungary, Iceland, Ireland, Israel, Italy, Japan, Korea, Latvia, Lithuania, Luxemburg, Malta, Mexico, New Zealand, North Macedonia, Norway, Poland, Portugal, Russian Federation, Serbia, Slovak Republic, Slovenia, Spain, Sweden, Switzerland, Netherlands, Türkiye, United Kingdom and the United States. For each country, the web pages of the respective ministries responsible for health and social care were searched using automatic translation provided by the web browser (Google Chrome; Google Translate). References to other institutions with delegated responsibilities for LTC systems or performance monitoring were also explored. Additionally, national statistics institutions were directly searched in each country for LTC-related data. In cases where information was inconclusive, additional web searches were conducted using terms “long-term care”, “long-term care quality indicators” in both the country’s main language and English, along with the country’s name. Information on quality monitoring provided by the Joint Report on Health care and Long Term Care Systems and Fiscal Sustainability [31] further guided the search for indicators used in EU countries. While it was not feasible to hand search all individual subnational jurisdictions within each country, a specific search was conducted for performance monitoring of LTC in subnational jurisdictions referenced in the literature.

### Expert consultation

The first round of web searches was performed between March and May 2022, during which a table was compiled with links to the data sources for each country. The Working Party on Health Care Quality and Outcomes, consisting of OECD member countries representatives, was consulted between May and September 2022. First, the preliminary results and the purpose of the study were presented at an in-person meeting to the members of the Working Party in Paris on 12 May 2022. After the meeting, a written request was sent to the members to review the identified information sources for their respective countries and provide corrections or additional information. Based on their feedback, the sources were updated and data on performance indicators for each country were extracted. The findings were presented to the same Working Party in May 2023, providing an additional opportunity for participating experts to offer feedback on the findings.

### Data extraction and analysis

Data extraction diverged from the planned model tables outlined in the registered protocol. Following full paper reviews, it became apparent that only a subset of papers provided detailed and comprehensive descriptions of national or subnational indicators or frameworks for performance monitoring of LTC suitable for direct extraction. Even in these cases, more updated sets of indicators were accessible through the websites of the organizations responsible for the monitoring. Consequently, our final selection of original research / scientific papers primarily facilitated the review of referenced LTC sets of indicators through grey literature.

The presentation of performance monitoring of LTC systems and services by national and subnational authorities is structured around each national or subnational jurisdiction. This contrasts with our initial expectation, outlined in the study protocol, of finding comprehensive frameworks for monitoring LTC systems and services on a national or subnational level. For each jurisdiction, we identified the responsible authority, the number of indicators being monitored, and the LTC settings these indicators apply to. Additionally, each indicator was categorized based on the specific structure, process or result that it aims to monitor (e.g., falls, malnutrition, polypharmacy, etc.).

## Results

**Fig. 1:**
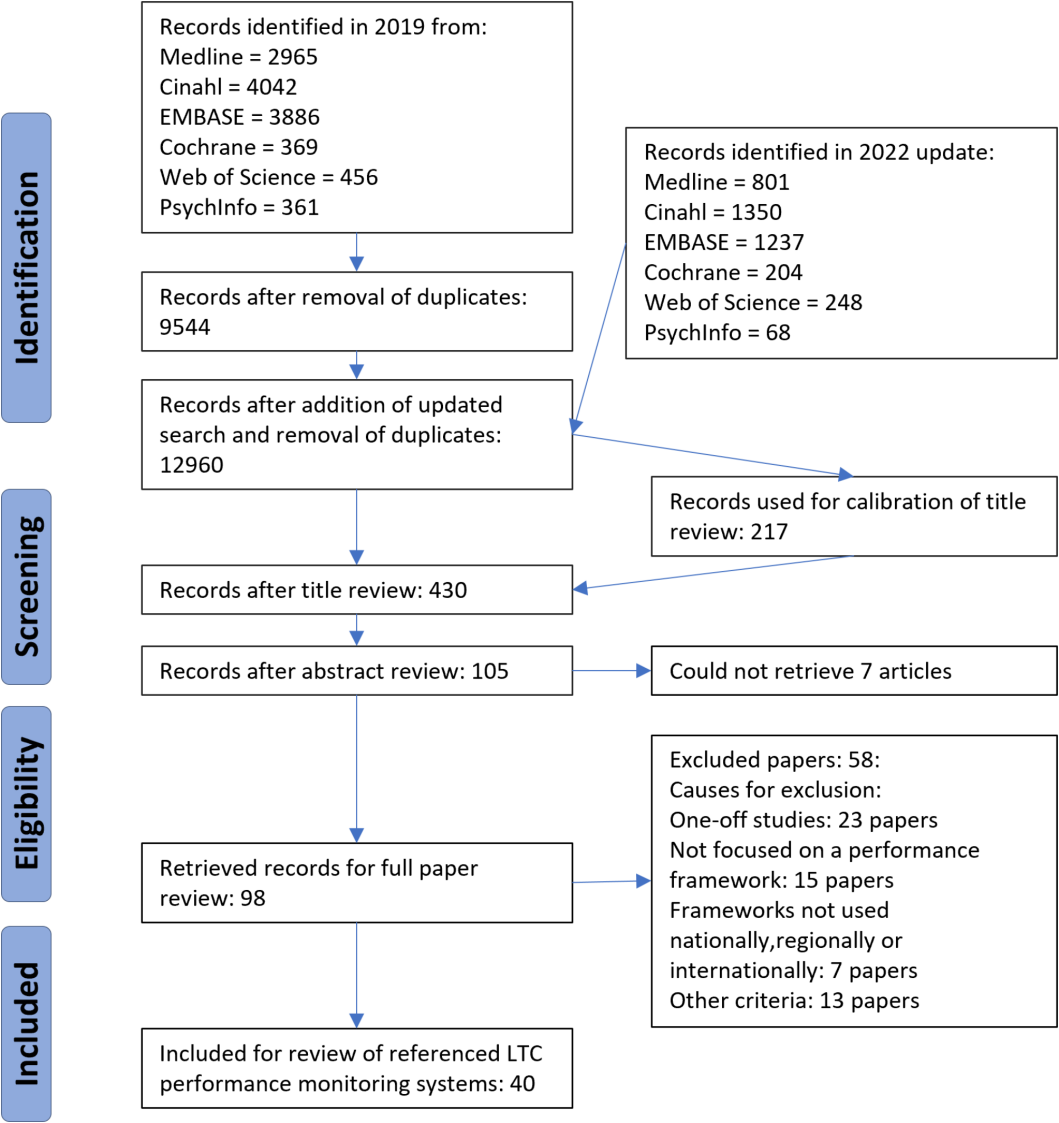
Flow chart of record reviewed

The initial scientific database search yielded 12,960 records after removal of duplicates. Following abstract screening, 98 papers were selected for full review. Following full paper reviews, fifty-eight papers were excluded, mostly because they focused on one-off studies or did not pertain to monitoring LTC systems and services at national, regional or international levels. Ten papers satisfied all inclusion and exclusion criteria. Additionally, 30 papers included specific references to performance monitoring of LTC nationally or subnationally. All of these papers (40 in total) were used to identify performance monitoring of LTC at national and subnational level (Fig. 1). In the last step of the literature review process, seven authors worked in pairs to assess the 98 papers in a full-text review. Initially, 57 papers were retained, though 17 of these involved initial disagreements on whether they met all inclusion and exclusion criteria or should be used for their references to LTC performance monitoring. All disagreements were resolved through discussion between the reviewing pairs.

The monitoring described in the identified papers are not necessarily a comprehensive and up-to-date description of how the performance of LTC systems and services is monitored in these jurisdictions. Hence, the papers were used as pointers to existing indicators used by national or subnational authorities to monitor LTC performance (Table 1). The actual indicators used to monitor LTC performance and their characteristics were extracted from the grey literature, in particular from web-based material.

**Table 1:** Overview of scientific papers referencing LTC performance monitoring by national or subnational authorities

Jurisdiction referenced in the papers	Identified papers
**Papers referencing performance monitoring in a single national and subnational jurisdiction**	
Australia	Lalic et al. (2016) [32] Cardona (2018) [33] Inacio et al. (2020) [34]
Belgium	De Schreye et al. (2020) [35]
Brazil	Fonseca de Oliveira et al. (2017) [36]
Canada (excluding specific papers on Ontario)	Mashouri et al. (2020) [37] Firbank (2012) [38] Fraser et al. (2017) [39]
Catalonia	García-Altés et al. (2021) [40]
England	Bulamu (2015) [41] Cook & Horrocks (2016) [42] Barron & West (2017) [43]
Iceland	Hjaltadottir et al. (2012) [44]
Korea	Lee & Shin (2019) [45]
Minnesota	Dongjuan et al. (2019) [46]
Netherlands	Triemstra et al. (2010) [47]
Ontario	Doran et al. (2009) [48] Wilkinson et al. (2019) [49]
Sweden	Lind et al. (2013) [50]
Switzerland	Favez et al. (2020) [51] Zuniga et al. (2019) [52]
Tuscany	Barsanti et al. (2021) [53]
Umbria	Montanari et al. (2018) [54]
United States (excluding specific papers on Minnesota)	Lenard & Shimshak (2009) [55] Castle (2009a) [56] Castle (2009b) [57] Lee (2009) [58] Rosati (2009) [59] Castle & Ferguson (2010) [60] DeLellis & Ozcan (2013) [61] Backhaus et al. (2014) [62] Dulal (2018) [63] Burgess et al. (2018) [64]
**Papers referencing performance monitoring in several national and subnational jurisdictions**	
Austria, Germany, Ireland, Netherlands, Sweden, UK and United States.	Du Moulin et al. (2010) [65]
Czechia, Finland, France, Germany, Israel, Italy, Netherlands and England.	Frijters et al. (2013) [66]
Czechia, Denmark, Finland, Germany, Italy and Netherlands	Foebel et al. (2015) [67]
Canada, Netherlands, Sweden, UK and United States.	Joling et al. (2018) [16]
Belgium, Canada, Denmark and Netherlands	Dequanter et al. (2020) [68]

Twelve papers referenced performance monitoring in the United States (30%), followed by Canada with five papers (12%), two of which were focused on the province of Ontario. Three papers (7%) referred to monitoring performance in England and Australia, two papers to Switzerland while other countries and regions were the focus of one paper each. Additionally, 5 papers (12%) were multi-country studies or reviews. A paper referenced performance monitoring in the Italian region of Umbria using the InterRAI instrument [69], however more detailed information on how the instrument is used to monitor performance could not be retrieved. Similarly, a paper referenced plans to monitor indicators in Brazil [36], but more detailed information on indicators used was not found.

The hand search of non-indexed grey literature including web-based materials related to 43 high- and middle-income countries lead to the identification of performance monitoring of LTC systems and services in 22 countries (Australia, Austria, Belgium, Canada, Chile, Denmark, France, Germany, Iceland, Ireland, Israel, Italy, Luxembourg, Netherlands, New Zealand, Norway, Portugal, Slovenia, Spain, Sweden, UK and United States). The list of resources identified through the hand search, describing performance monitoring of LTC systems and services in each country was submitted to member country representatives of the Working Party on Health Care Quality and Outcomes of the OECD. Responses were obtained from 18 countries. Five of these responses confirmed the accuracy of the resources identified, responses from 11 countries provided additional resources on performance monitoring of LTC. Two responses (from Korea and Switzerland) provided information on previously unidentified national performance monitoring of LTC systems and services.

The papers identified in the scientific literature review, which reference national level performance monitoring of LTC, did not provide additional information. However, references in the papers to subnational performance monitoring allowed the identification of LTC indicators used in subnational jurisdictions of Catalonia (Spain), Minnesota (United States), Ontario (Canada), Scotland (UK) and Tuscany (Italy).

### Indicators used for LTC performance monitoring

Based on scientific and grey literature review, and expert consultation, indicators specifically related to LTC were identified as integral components of regular performance monitoring across 29 national and subnational jurisdictions. Performance monitoring of LTC systems and services through indicators can be structured in different ways. Australia for example has a comprehensive framework called the National Aged Care Mandatory Quality Indicator Program. Within this program all national performance indicators related to LTC are collected. On the other hand, in the United States there are different frameworks to monitor the performance of nursing homes and skilled nursing facilities, home care agencies and LTC hospitals. Furthermore, in countries such as Canada, Sweden, Italy and Norway, LTC-related performance indicators are often integrated into broader health and/or social care monitoring frameworks. For example, in Italy national performance monitoring of LTC is part of the regulation called “New guarantee system for health care monitoring”, which covers the whole health sector [70].

Table 2 provide a description of performance monitoring of LTC systems and services in 29 national and subnational jurisdictions where such monitoring was identified. For each jurisdiction, we identified the authority responsible for monitoring, and the number of indicators monitored. Subnational monitoring reported in Table 2 is limited to subnational specificities, i.e. it should be understood as an addition to national performance monitoring, if present. For example, the performance monitoring of LTC in Canada also applies to the province of Ontario. Performance monitoring in Ontario reported in Table 2 refers to the additional monitoring, specific to Ontario.

**Table 2:** Overview of retrieved LTC performance monitoring approaches by jurisdiction

National/ subnational jurisdiction	Short description of the LTC performance monitoring framework	Responsible authority	Total number of indicators monitored	Settings to which it is applied	Information sources
Australia	The National Aged Care Mandatory Quality Indicator Program established a national framework for monitoring long-term care, currently in residential settings.	Department of Health and Aged Care of the Government of Australia	28	Long-term care facilities	Department of Health and Aged Care (2022) [71]
Austria	Long term care performance is monitored within a broader framework called “Outcome-Messung im Gesundheits- wesen” [Outcome measurement in healthcare]. The framework has a section called “long-term care and support”, where indicators relevant to long-term care are collected.	Federal Ministry for Social Affairs, Health, Care and Consumer Protection	5	Home care, long-term care facilities, recipients of nursing allowance (in all settings)	Bundesministerium für Soziales Gesundheit Pflege und Konsumentenschutz (2021) [72]
Belgium	The indicators are part of the domain called “care for the elderly”, which is part of the Health System Performance Assessment framework used in Belgium	The data is made available by the portal Belgium.be. Data provided by several institutions.	15	Home care, rest homes for the elderly (MRPA) and rest and care homes (MRS)	healthybelgium.be portal [73]
Canada	Long-term care is monitored with the support of InterRAI instruments both in long-term care facilities and related to home care. Data are published on the “Your health system” portal together with other health care performance related data, as part of a comprehensive measurement framework for the performance of the health care system.	Canadian Institute for Health Information (CIHI)	40	Long-term care facilities, home care	Your Health System portal [74], Indicator library [75], the Performance measurement framework [76]
Catalonia	Within the quality monitoring system in health care, so called “intermediate care” is reported. It includes palliative care services, long stay units and home care, among other types of services.	Health Quality and Assessment Agency of Catalonia (AQuAS)	41	Long stay units, palliative care, home care	Observatori del Sistema de Salut de Catalunya (2023) [77]
Chile	Within the national set of performance indicators (related to all government activities, not only health or social care) there is a section dedicated to services for older persons.	National service for older persons (Servicio nacional del adulto mayor - SENAMA)	4	Nursing homes, day care centres, users of SENAMA services	Dirección de Presupuestos Gobierno de Chile (2020) [78]
Denmark	The annual report is published under the heading Social benefits for senior citizens. It includes a number of indicators that refer to the care provided at municipal level to older persons.	Data published by Statistics Denmark	17	Home care and nursing, nursing homes, exercise services, rehabilitation, preventative home visits	Social benefits for senior citizens, statistical presentation portal [79]
England	The Adult Social Care Outcomes Framework (ASCOF) includes a list of indicators related to long term care. The framework aims at monitoring recipients of adult social care services, which include older persons and other in need of social care and support.	Department of Health and Social Care	20	Recipients of adult social care services, including residential and home care services	Department of Health and Social Care (2018) [80]
France	National regulation sets a list of indicators to monitor the performance of long-term care facilities (établissement d’hébergement pour personnes âgées dépendantes - EHPAD).	National solidarity fund for autonomy (Caisse nationale de solidarité pour l’autonomie - CNSA)	5	Long-term care facilities (établissement d’hébergement pour personnes âgées dépendantes - EHPAD)	Regulation on indicators’ methodology (2022) [81]
Germany	Long-term care indicators are set in the regulation as an annex to the standards and principles for quality, quality assurance and presentation as well as for the development of internal quality management in accordance with Section 113 of the Eleventh Book of the Social Code (SGB XI) in inpatient care.	The agreement based on the regulation that sets the indicator is signed by key stekholders in the long-term care system in Germany.	15	Long-term care facilities	Appendix 2 of the standards and principles of quality, quality assurance and presentation [82]
Iceland	Performance monitoring of nursing homes is based on InterRAI instruments and performance indicators used within InterRAI. Data are published as a dashboard.	Office of the national medical examiner	22	Long-term care facilities	Nursing homes quality indicators’ portal [83]
Ireland	Within the National Service Plan, key performance indicators are published by type of service. Among the services, Older Persons Services are related to long-term care. Furthermore, the Health Information and Quality Authority publishes data on so called “notifications” which have developed into indicators, including some related to Older Persons Services.	Health Service Executive	30	Home care and residential care	Health Service Executive (2022) [84]
Israel	Within the National program for quality indicators there is section related to geriatric hospitals, with indicators relevant to long-term care.	Ministry of Health of Israel	18	Long term geriatric wards, long term mechanical ventilation wards, rehabilitation wards and acute care geriatric wards.	Quality & Safety Division Health Services Research Department (2022) [85]
Italy	Within the national regulation called “New guarantee system for health care monitoring” there is a set of indicators, which include performance indicators in long-term care. Long-term care indicators are not grouped separately. They are found under the heading “District assistance”.	Ministry of Health of Italy	2	Nursing homes, home care	Decree of the Ministry of Health of 12 March 2019 New guarantee system for health care monitoring [70, 86]
Korea	Regular nursing hospital inpatient benefit adequacy evaluations include performance indicators for long-term care. The information refers to so called long-term care hospitals.	Health Insurance Review and Assessment Service (HIRA)	14	Long-term care hospitals	Periodic evaluation of long-term care services [86]
Luxembourg	Within the legislation of Luxembourg there is an aid and care framework, which addresses indicators in the area of long-term care. The data are published biannually.	State Office for Assessment and Monitoring of the long-term care insurance	7	All persons who have a needs of more than 3,5 h/week of help in ADL, regardless of age are included in the monitoring. This includes nursing homes, day care, and supported living facilities.	State Office for Assessment and Monitoring of the long-term care insurance (2020) [87]
Minnesota	Nursing homes report card monitors short stay and long stay nursing homes. Reported indicators are composite indicators from several data sources.	Minnesota Department of Human Services and Minnesota Department of Health	8	Long stay nursing homes	Minnesota Department of Human Services, Minnesota Department of Health, (2023) [88]
Netherlands	Quality monitoring is based on two frameworks, which differ by the settings to which they apply: a Nursing home care quality framework and a Quality framework for community nursing.	The Nursing home care quality framework is administered by the Healthcare Institute of the Netherlands (Zorginstituut Nederland). The Quality framework for community nursing is administered by a consortium, which includes the Netherlands Patient Federation and several trade organizations.	24	Nursing homes, home care	Zorginstituut Nederland (2022a) [89]; Zorginstituut Nederland (2022b), [90]; Quality Framework for Community Nursing portal [91]; Actiz et al.(2022) [92]
New Zealand	National InterRAI Quality Indicators are a set of performance indicators related to long-term care facilities, based on the InterRAI instruments and indicators developed within the international consortium	interRAI Services, a business unit within Te Whatu Ora - Health New Zealand	31	Long-term care facilities	InterRAI New Zealand, TAS Kahui tuitui tangata (2018) [93]
Norway	The National Quality Framework includes health and care services. The indicators are grouped by topic. There is no long-term care or old person care topic. Most indicators related to long-term care can be found in the indicator topic “municipal health and care services”.	Directorate of Health (Helsedirektoratet)	13	Nursing homes and home health services	National Quality indicators portal [94]
Ontario	A set of quality indicators has been established with a dedicated section to home care. The home care set of indicators is based on a several data source, including those provided with the instrument InterRAI Home Care.	Health Quality Ontario	37	Nursing homes and home care	Ontario Health Indicator Library portal [95]
Portugal	Performance indicators related to long-term care can found within the “Transparency” portal of the national health service. The indicators relevant to LTC are related to the services provided by the National Integrated Continuing Care Network. Within the network there are stationary and home services provided.	National Health Service (SNS) of Portugal	2	Residential and home care settings within the National Integrated Continuing Care Network	Transparencia portal [96]
Scotland	The Care Inspectorate regularly publishes data about the quality of registered services, presented as the summary of grades on each of the care standards. The summary represents the percentage of registered services with a particular grade on a particular standard. Services include adult placement services and care home services for older people.	Care Inspectorate Scotland	5	Long term care facilities, home care	Quarterly Statistical Summary Reports portal [97]
Slovenia	A set of quality indicators supported by contextual indicators for nursing homes has been established by the Ministry of Health. Indicators’ results have not yet been published.	Ministry of Health	5	Nursing homes	Bolčević et al. (2021) [98]
Spain	A report discussing monitoring the quality of care for older persons identifies 3 indicators, which are regularly monitored and related to LTC. They are not part of a broader monitoring framework.	State association of Directors and Managers of Social Services	3	Nursing homes, home care	Davey (2021) [99]
Sweden	National data on the performance of health and social care is published on the “open comparisons” subpage of Socialstyrelsen. The indicators’ section most relevant to long term care is “social services and municipal health care - elderly care”. The perspective of the authority is to monitor the quality of care for older persons at the level of the municipality, rather than the quality of care in a particular long-term care setting.	National Board of Health and Welfare (Socialstyrelsen)	46	Home care, special housing for older persons, general population	Open comparisons portal [81, 100]
Switzerland	There is a federally agreed set of indicators called “Medical quality indicators” related to Medico-social establishments (EMS), which accommodate elderly people requiring long-term care.	Federal Office of Public Health (OFSP)	6	Long-term care facilities	Federal Office of Public Health (2021) [101]
Tuscany	Voluntary performance evaluation system developed by the Region of Tuscany. Most data are collected annually, some data are collected every two years.	Region of Tuscany	107	Nursing homes	Barsanti et al. (2020) [102]
USA	There are different performance monitoring systems for different types of services. Short stay and long stay quality measures are indicators related to nursing homes and so called “skilled nursing facilities”. Furthermore, there are process and outcomes quality measures related to services provided by home health agency. Finally, there is an established set of indicators related to long-term care hospitals. The indicator findings feed into the Care Compare portal of Medicare.gov, where each group of service providers has a separate subsection.	the Center for Medicare and Medicaid Services (CMS)	49	Skilled nursing facilities, nursing homes, long-term care hospitals, home care	Measures portals for Skilled nursing facilities [103], Home care [104] and long term care hospitals [105]

*Abbreviations*: *ADL* Activities of daily living

We extracted a total of 620 indicators from the resources on performance monitoring of LTC in the 29 national and subnational jurisdictions identified (Additional file 4). 438 of these indicators were assigned specific domains by the monitoring authority, while the remaining 182 indicators were not assigned a domain. Some monitoring authorities identified domains of quality used by the World Health Organization, such as safety and effectiveness For example, indicators related to safety were identified in Israel, the Netherlands, Canada, Belgium, Sweden, and Catalonia. Effectiveness was used as a domain for performance monitoring in Canada, the United States, and Catalonia. In contrast, other monitoring authorities adopted domains with a narrower scope. In Israel, for example, domains such as diet, pain assessment, and drug therapy management are used, while in Denmark and Catalonia broader categories like “background indicators” and “general data” are employed. Austria, Germany, and England directly link indicator domains to the goals of their LTC systems, such as Germany’s “Quality Area 1: maintaining and promoting independence”.

**Fig. 2:**
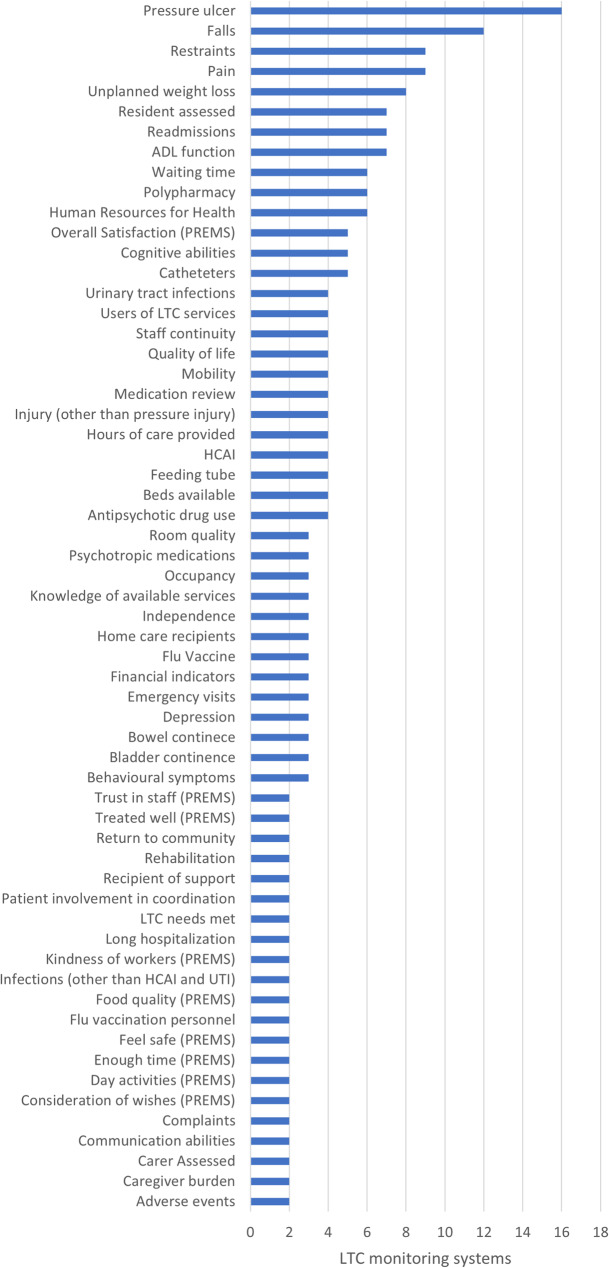
Structures, processes, and results monitored by two or more jurisdictions (*N*=29). List of abbreviations: ADL– Activities of daily living; HCAI– Health care associated infections; PREMS– Patient reported experience measures; UTI– Urinary tract infections

These 620 indicators collectively monitor 188 different structures, processes, and results related to LTC. Jurisdictions most often differed in the specific definitions of indicators related to the same event or process. Sometimes several indicators within one jurisdiction refer to the same event or process (e.g., falls and falls that lead to emergency room visits, hospitalizations, or deaths). We counted each monitored event and process frequency across jurisdictions, noting that pressure ulcers were the most commonly monitored (in 16 jurisdictions), followed by falls (12 jurisdictions), use of restraints, and monitoring of pain (9 jurisdictions each). Figure 2 reports all structures, processes, and results monitored by more than one jurisdiction.

**Fig. 3:**
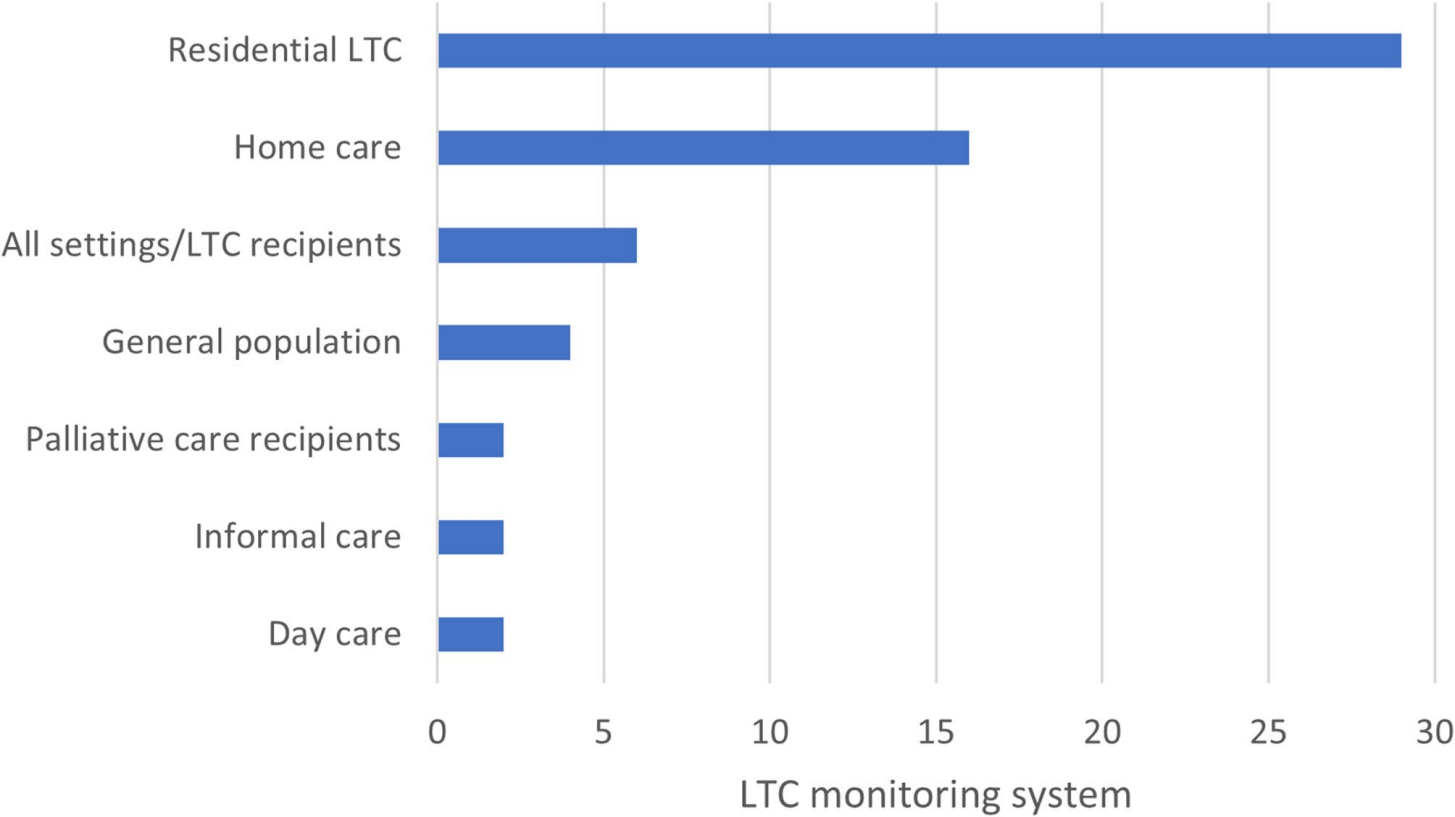
Frequency of settings being monitored by national and subnational jurisdiction (*N*=29)

Indicators were grouped based on the LTC settings that they monitor. Indicators monitoring residential LTC services include all settings providing overnight stays. These include nursing homes, and also LTC hospitals or wards monitored in Korea and Israel, as well as special housing for the elderly provided in Sweden. Home care indicators sometimes overlap with community nursing services, making it challenging to distinguish between them. Therefore, these were grouped together. Additionally, indicators relevant to all LTC services within a particular jurisdiction, regardless of setting, were categorized under “LTC recipients/multiple settings”. In all cases these setting include residential LTC and home care. The “general population” group refers to indicators that aim to monitor LTC indirectly, by observing phenomena in the general population, such as 30-day readmissions to hospitals among patients aged 65 years and older. Figure 3 shows that all jurisdictions include indicators relevant to residential LTC services. Additionally, indicators for home care were identified in 16 jurisdictions, accounting for 55% of the total.

## Discussion

In our comprehensive review covering 43 countries, we identified LTC performance indicators in 24 of them, including 6 subnational jurisdictions. The UK was counted as one country, with performance monitoring found in two subnational jurisdictions—England and Scotland. This encompassed a total of 620 indicators, monitoring 188 structures, processes, and results related to LTC. Predominantly monitored structures, processes, and results included pressure ulcers, falls, use of restraints, and pain assessments. Across all jurisdictions with identified indicators, residential LTC facilities was consistently monitored, with home care also included in 16 out of 29 jurisdictions. The domains that were most frequently identified relate to the domains of quality used by the World Health Organization [106], safety in particular. However, the classification of domains varied considerably among jurisdictions, with 182 indicators not assigned to any specific domain. This highlights the need for a more standardized approach to categorizing and reporting LTC performance indicators globally.

Our findings on the most frequently reported indicators are consistent with those of recently published papers with a similar focus [22, 107]. Even though Osinska et al. [22] focused on residential LTC, they also found pressure ulcers, falls and restraints to be the most frequently monitored indicators. Loureiro and Martin [107] also focused on residential LTC and noted the frequent use of pressure ulcers indicators, as well as indicators on psychotropic drug use and functional performing. Notably, their review highlighted the relative abundance of studies based in the United States in the papers they reviewed [107]. Both reviews had some additional restrictions on eligible performance monitoring that we did not employ (e.g. publicly reported indicators or those based on secondary use of data sources). Consequently, we were able to retrieve a larger number of LTC indicators.

Common indicators tend to focus on medical or health related aspects of care, pointing to future challenges in internationally agreeing on indicators, particularly in areas related to social care, users’ experiences and users’ reported outcomes.

A report published by the OECD in 2013 [6] mentioned six performance frameworks for LTC in six jurisdictions. While direct comparisons are not possible, as the scope and purpose of the OECD publication were different from our study, the information suggests that LTC performance monitoring has been developing internationally over the past decade. The variety of domains and indicators monitored among jurisdictions may be a consequence of the variability in how LTC is organized in each jurisdiction. The short indicators descriptions reveal that even when the same event or process (such as pressure ulcers) is monitored, the actual methodologies differ, thus posing additional challenges for developing a common set of internationally comparable indicators.

The designation of domains in LTC that are monitored through a set of indicators (e.g. patient safety, patient-centeredness) relates the indicators to the broader policy goals or performance objectives set out by stakeholders [108, 109]. This is evident when the performance domains are actually policy objectives, as in Austria, Germany and England. However, indicators were often not assigned to specific domains, and in other cases, domains did not constitute a consistent and comprehensive approach to relate indicators to LTC goals or objectives. For example, in Portugal we found 2 nationally monitored indicators (on falls and pressure ulcers) which were not assigned a domain. In Spain the three indicators monitored nationally are also not assigned a domain. This suggests that feasibility issues, such as the availability of data sources, may have constrained the choice of indicators monitored.

The LTC performance indicators were sometimes distributed across different indicator sets (e.g. home care as separated from LTC in the Netherland and Canada) and sometimes part of a broader monitoring approach (e.g. the National Quality Framework for health and care services in Norway or the government performance indicators used in Chile). This suggests that not all countries or regions have identified LTC for older people as an entity that requires separate and comprehensive policy attention or governance.

### Strengths and limitations

The broad scope of the study is a major strength. We conducted a thorough search for LTC performance monitoring across 43 countries, encompassing diverse settings relevant to older people. This broad approach ensures a comprehensive exploration of international practices in LTC monitoring. Moreover, our focus on high- and middle-income countries enhances the study’s robustness by targeting regions with established infrastructures for data collection and reporting on performance indicators [110, 111]. This emphasis increases the likelihood of identifying comprehensive and comparable LTC performance indicator sets.

In addition to our broad geographical coverage, we employed multiple methodologies to maximize comprehensiveness. These included rigorous web searches, supplemented by a comprehensive literature review and expert consultations. These efforts were instrumental in minimizing the possibility of overlooking significant efforts in LTC monitoring by national or subnational authorities. Despite our comprehensive approach, our study has several limitations. The scientific literature search focused on indicator frameworks, which may lead to omissions of some performance monitoring efforts. Furthermore, we note a relatively high level of initial disagreements between reviewers, despite the calibration process. The reliance on web searches does not guarantee completeness. Furthermore, the web searches were conducted by one author (MP), using automatic translation tools where necessary, which may have led to the omission of indicator sets due to language barriers or search term variations. A lack of detailed knowledge about each performance monitoring effort is also a limitation. To mitigate this, we sought validation from area experts in several countries. Similarly, as data extraction may be affected by the same limitation, our analysis focused on basic indicator characteristics to ensure consistency. Furthermore, while our study identified a broad array of LTC performance indicators, we did not conduct a detailed appraisal of the jurisdictions’ approaches or their indicator sets. This limitation stems from our study’s primary objective, which focused on identifying feasible and relevant LTC monitoring indicators across a wide range of high- and middle-income countries.

### Implications

Our study contributes valuable insights into the current state of LTC performance monitoring globally. By identifying the commonalities and differences in monitored indicators, our findings can inform policymakers and stakeholders about the key areas needing standardisation. The study underscores the importance of developing consistent domains and methodologies to enhance the comparability of LTC performance data internationally. Future research should focus on exploring the feasibility of implementing a unified set of performance indicators across different LTC systems, while also considering the appropriateness of the indicators in the contexts where they would be used.

## Conclusions

Our study identified 29 national or subnational jurisdictions currently monitoring a total of 620 LTC performance indicators. Among these, the most frequently used indicators relate to pressure ulcer, falls, use of restraints and pain assessments. Despite this commonality, there is notable inconsistency among countries regarding how LTC performance indicators are structured across settings and domains. All jurisdictions monitor performance related to residential LTC and more than half also monitor LTC services in home care settings.

The variety of LTC systems across jurisdictions is reflected in the variety of performance monitoring approaches. This diversity poses additional challenges to establishing a set of internationally comparable performance indicators that can facilitate mutual learning. Some structures, processes, and results are monitored frequently, but the specific indicators definitions tend to differ between jurisdictions. Precise methodological specifications and risk adjustments will be necessary to ensure that populations and LTC services can be effectively compared across jurisdictions.

## Supplementary Information

Additional file 1. Preferred Reporting Items for Systematic reviews and Meta-Analyses extension for Scoping Reviews (PRISMA-ScR) Checklist. Compiled checklist.

Additional file 2. Medline (OVID) Search Strategy. An example of the search strategy used for the scientific database review.

Additional file 3. Inclusion, exclusion criteria and definitions. The list of criteria used to screen the papers retrieved.

Additional file 4. Performance indicators for monitoring LTC. The full list of 620 indicators extracted from 29 national and subnational jurisdictions.

## Data Availability

All the data and materials used are cited in-text and included in the references.

## Abbreviations

ADL: Activities of daily living
EOLC: End of life care
ER: Emergency room
GP: General practitioners
HbA1cC: Glycated haemoglobin
HCAI: Health care associated infections
HRH: Human resources for health
LTCF: Long term care facilities
PREMS: Patient reported experience measures
QoL: Quality of life
UTI: Urinary tract infections
