# Supplementary material for: Performance indicators on long-term care for older people in 43 high- and middle-income countries: literature review, web search and expert consultation
Source: BMC Health Serv Res. 2025 Mar 28;25:460. doi: 10.1186/s12913-025-12573-4 (PMC11951636; doi:10.1186/s12913-025-12573-4)
Supplement: Supplementary file 2 — Additional file 2. Medline (OVID) Search Strategy. An example of the search strategy used for the scientific database review. [file 12913_2025_12573_MOESM2_ESM.docx]

**Medline (OVID) Search Strategy**

Results of the search performed on 4 April 2019

| Sequential number | Query | No of items retrieved |
| --- | --- | --- |
| 1 | "Organization and Administration"/og, st, sn [Organization & Administration, Standards, Statistics & Numerical Data] | 169 |
| 2 | exp Clinical Governance/og, st, sn [Organization & Administration, Standards, Statistics & Numerical Data] | 176 |
| 3 | "Quality of Health Care"/og, st, sn [Organization & Administration, Standards, Statistics & Numerical Data] | 12804 |
| 4 | Quality Assurance, Health Care/og, st, sn [Organization & Administration, Standards, Statistics & Numerical Data] | 13926 |
| 5 | exp Guidelines as Topic/st [Standards] | 9736 |
| 6 | Quality Improvement/og, st, sn [Organization & Administration, Standards, Statistics & Numerical Data] | 5111 |
| 7 | Health Services Accessibility/og, st, sn [Organization & Administration, Standards, Statistics & Numerical Data] | 19312 |
| 8 | Health Care Rationing/og, st, sn [Organization & Administration, Standards, Statistics & Numerical Data] | 2128 |
| 9 | Health Equity/og, st, sn [Organization & Administration, Standards, Statistics & Numerical Data] | 174 |
| 10 | Health Expenditures/st, sn [Standards, Statistics & Numerical Data] | 5726 |
| 11 | exp Efficiency, Organizational/st, sn [Standards, Statistics & Numerical Data] | 1345 |
| 12 | Total Quality Management/og, st, sn [Organization & Administration, Standards, Statistics & Numerical Data] | 5134 |
| 13 | 1 or 2 or 3 or 4 or 5 or 6 or 7 or 8 or 9 or 10 or 11 or 12 | 71365 |
| 14 | "Organization and Administration"/ | 14574 |
| 15 | exp Clinical Governance/ | 487 |
| 16 | "Quality of Health Care"/ | 69045 |
| 17 | Quality Assurance, Health Care/ | 54762 |
| 18 | exp Guidelines as Topic/ | 149684 |
| 19 | Quality Improvement/ | 19687 |
| 20 | Health Services Accessibility/ | 68307 |
| 21 | Health Care Rationing/ | 11152 |
| 22 | Health Equity/ | 722 |
| 23 | Health Expenditures/ | 18534 |
| 24 | exp Efficiency, Organizational/ | 21071 |
| 25 | Total Quality Management/ | 12376 |
| 26 | 14 or 15 or 16 or 17 or 18 or 19 or 20 or 21 or 22 or 23 or 24 or 25 | 405333 |
| 27 | Models, Organizational/ | 18331 |
| 28 | Models, Theoretical/ | 142641 |
| 29 | "Process Assessment (Health Care)"/ | 4384 |
| 30 | Program Evaluation/ | 59008 |
| 31 | exp "Standard of Care"/ | 3063 |
| 32 | 27 or 28 or 29 or 30 or 31 | 224595 |
| 33 | 26 and 32 | 20467 |
| 34 | "Outcome and Process Assessment (Health Care)"/ | 25663 |
| 35 | "Outcome Assessment (Health Care)"/ | 67082 |
| 36 | exp Quality Indicators, Health Care/ | 19335 |
| 37 | exp Benchmarking/ | 12505 |
| 38 | Patient Outcome Assessment/ | 4054 |
| 39 | Patient Reported Outcome Measures/ | 2882 |
| 40 | 34 or 35 or 36 or 37 or 38 or 39 | 125553 |
| 41 | 13 or 33 or 40 | 200892 |
| 42 | ((performance or quality or indicator? or standard?) adj2 (framework? or model? or frame? or structure? or scheme? or Schema)).ti,ab. | 23901 |
| 43 | ((evaluation? or assessment? or monitoring or effectiveness or safety or coordination or integration or Access$ or expenditure?) adj2 (framework? or model? or frame? or structure? or scheme? or Schema)).ti,ab. | 42225 |
| 44 | ((responsiveness or patient center$ or continuity or patient experience? or patient reported experience? or patient reported outcome? or PREMS or PROMS) adj2 (framework? or model? or frame? or structure? or scheme? or Schema)).ti,ab. | 926 |
| 45 | 42 or 43 or 44 | 65233 |
| 46 | 41 or 45 | 263082 |
| 47 | exp Long-Term Care/ | 24916 |
| 48 | exp Health Services for the Aged/ | 17073 |
| 49 | exp Assisted Living Facilities/ | 1276 |
| 50 | exp Group Homes/ | 945 |
| 51 | exp Halfway Houses/ | 1050 |
| 52 | exp Homes for the Aged/ | 13224 |
| 53 | exp Nursing Homes/ | 37442 |
| 54 | Residential Facilities/ | 5261 |
| 55 | exp AGED/ | 2923948 |
| 56 | exp GERIATRICS/ | 29137 |
| 57 | exp Geriatric Nursing/ | 13386 |
| 58 | exp Geriatric Dentistry/ | 980 |
| 59 | exp Geriatric Psychiatry/ | 2284 |
| 60 | exp Geriatric Assessment/ | 25084 |
| 61 | 55 or 56 or 57 or 58 or 59 or 60 | 2944952 |
| 62 | exp Home Care Services/ | 45420 |
| 63 | 61 and 62 | 15722 |
| 64 | 47 or 48 or 49 or 50 or 51 or 52 or 53 or 54 or 63 | 95658 |
| 65 | long term care.ti,ab. | 18717 |
| 66 | nursing home?.ti,ab. | 27975 |
| 67 | home? for the elderly.ti,ab. | 1392 |
| 68 | home? for the aged.ti,ab. | 1285 |
| 69 | (home adj2 care).ti,ab. | 24166 |
| 70 | (home adj2 health service?).ti,ab. | 628 |
| 71 | (community adj2 care).ti,ab. | 11395 |
| 72 | (community adj2 health service?).ti,ab. | 2823 |
| 73 | (nursing adj2 care).ti,ab. | 30771 |
| 74 | (nursing adj2 service?).ti,ab. | 5482 |
| 75 | (institutional adj2 care).ti,ab. | 3073 |
| 76 | (institutional adj2 health service?).ti,ab. | 33 |
| 77 | (residential adj2 care).ti,ab. | 4216 |
| 78 | (residential adj2 health service?).ti,ab. | 25 |
| 79 | (nursing adj2 facility).ti,ab. | 2249 |
| 80 | (residential adj2 facility).ti,ab. | 613 |
| 81 | 69 or 70 or 71 or 72 or 73 or 74 or 75 or 76 or 77 or 78 or 79 or 80 | 78074 |
| 82 | elderly.ti,ab. | 226455 |
| 83 | frail.ti,ab. | 10025 |
| 84 | (old person? or old patient?).ti,ab. | 30581 |
| 85 | geriatric patient?.ti,ab. | 6924 |
| 86 | extended.ti,ab. | 245841 |
| 87 | social care.ti,ab. | 5349 |
| 88 | 81 or 87 | 82740 |
| 89 | 82 or 83 or 84 or 85 or 86 | 507677 |
| 90 | 88 and 89 | 7246 |
| 91 | 65 or 66 or 67 or 68 or 90 | 50390 |
| 92 | 64 or 91 | 114208 |
| 93 | 46 and 92 | 7364 |
| 94 | 93 | 7364 |
| 95 | limit 94 to yr="2009 -Current" | 2965 |
