# Supplementary material for: Performance indicators on long-term care for older people in 43 high- and middle-income countries: literature review, web search and expert consultation
Source: BMC Health Serv Res. 2025 Mar 28;25:460. doi: 10.1186/s12913-025-12573-4 (PMC11951636; doi:10.1186/s12913-025-12573-4)
Supplement: Supplementary file 3 — Additional file 3. Inclusion, exclusion criteria and definitions. The list of criteria used to screen the papers retrieved. [file 12913_2025_12573_MOESM3_ESM.docx]

**Inclusion, exclusion criteria and definitions**

3 Apr 2019, numbered 23 July 2019

## Inclusion criteria

1. The paper is focused on a **description of a performance framework**.
2. The performance framework includes **performance indicators or measurable/verifiable standards**
3. The subject of the performance framework is:
   1. **long-term care** (system or services) or
   2. particular long-term care **settings** *(N.B. There is no standardized, generally agreed upon classification of long-term care services or settings. Common distinctions include care provided at home, i.e. the usual place of residence, by either informal carers such as family members, or health professionals, care provided in the community for example at day centers, and care provided in a residential setting, which includes nursing homes, where residents share common spaces, if not the room, and assisted living facilities, where residents have their individual living spaces but are supported by professionals at the place of residence for activities of daily living*)(4); 2013 Congressional budget office report; 2016 CDC report on LTC providers
4. particular **dimension** of long-term care (e.g. access, safety, effectiveness,…)
5. particular **aspect of the long-term care system or services**, such as palliative care
6. Broader frameworks, that include LTC will be included if **LTC is recognized as a separate entity** (if the part referring to long-term care can be extracted)
7. Frameworks must be **regional/national/international**. Proposed frameworks, if identified, will be included.
8. Frameworks must **focus on older people** or include the older population as a distinct group.
9. The paper is a scientific original research paper (including reviews, excluding commentaries, editorials and non-peer-reviewed publications)

## Exclusion criteria

1. Frameworks on a particular disease or condition (e.g. falls, delirium), are excluded, except for Dementia, because it is mainly tackled in the context of long-term care.
2. Frameworks on a particular intervention or service, are excluded
3. Frameworks that do not focus on long-term care for the elderly (as opposed to other groups, who might need long-term care) are excluded
4. Frameworks used at the level of a single organization or local health authority will not be included.
5. Frameworks (or model) for cost effectiveness and other econometric analysis are not included, unless they are used nationally or regionally as regular (repeat) measures of performance of a health system, health setting or health service.
6. Single indicators (e.g. composite indicators based on an underlying model or framework) will not be included, unless they are part of an existing performance framework
7. One-off evaluations (even if national and include a framework and indicators) are not included

**Concept definitions**

Definition of “**long-term care”**

“Long-term care (LTC): is defined as a range of services required by persons with a reduced degree of functional capacity, physical or cognitive, and who are consequently dependent for an extended period of time on help with basic activities of daily living (ADL). This “personal care” component is frequently provided in combination with help with basic medical services such as “nursing care” (help with wound dressing, pain management, medication, health monitoring), as well as prevention, rehabilitation or services of palliative care. Long-term care services can also be combined with lower level care related to “domestic help” or help with instrumental activities of daily living (IADL)”(3).

Definition of “**elderly**”:

We set the threshold at 65 years or older, so age groups up to 64 years would not be considered elderly. However, the sources retrieved will be included in this scoping review, if they clearly focus on the elderly population, as defined by the retrieved publication or website. Only if the population will not be identified as old or elderly, but rather age groups will be provided, than the age defined as “elderly” will be used as the guidance to decide on which publications to include.
